# Chikungunya virus infectivity, RNA replication and non-structural polyprotein processing depend on the nsP2 protease’s active site cysteine residue

**DOI:** 10.1038/srep37124

**Published:** 2016-11-15

**Authors:** Kai Rausalu, Age Utt, Tania Quirin, Finny S. Varghese, Eva Žusinaite, Pratyush Kumar Das, Tero Ahola, Andres Merits

**Affiliations:** 1 Institute of Technology, University of Tartu, Tartu, Estonia; 2 Department of Food and Environmental Sciences, University of Helsinki, Helsinki, Finland

## Abstract

Chikungunya virus (CHIKV), genus *Alphavirus,* family *Togaviridae,* has a positive-stand RNA genome approximately 12 kb in length. In infected cells, the genome is translated into non-structural polyprotein P1234, an inactive precursor of the viral replicase, which is activated by cleavages carried out by the non-structural protease, nsP2. We have characterized CHIKV nsP2 using both cell-free and cell-based assays. First, we show that Cys478 residue in the active site of CHIKV nsP2 is indispensable for P1234 processing. Second, the substrate requirements of CHIKV nsP2 are quite similar to those of nsP2 of related Semliki Forest virus (SFV). Third, substitution of Ser482 residue, recently reported to contribute to the protease activity of nsP2, with Ala has almost no negative effect on the protease activity of CHIKV nsP2. Fourth, Cys478 to Ala as well as Trp479 to Ala mutations in nsP2 completely abolished RNA replication in CHIKV and SFV *trans-*replication systems. In contrast, *trans*-replicases with Ser482 to Ala mutation were similar to wild type counterparts. Fifth, Cys478 to Ala as well as Trp479 to Ala mutations in nsP2 abolished the rescue of infectious virus from CHIKV RNA transcripts while Ser482 to Ala mutation had no effect. Thus, CHIKV nsP2 is a cysteine protease.

Chikungunya virus (CHIKV) belongs to genus *Alphavirus* (family *Togaviridae*). It is transmitted by *Aedes* mosquitoes and has caused several massive outbreaks since 2004^1^. CHIKV has a positive-strand RNA genome approximately 12 kb in length. A large 5′ open reading frame (ORF), which covers 2/3 of the viral genome, is translated directly from the viral genomic RNA and encodes for a non-structural (ns) polyprotein designated as P1234. The second ORF encodes for the precursors of viral structural proteins and is translated from a specific subgenomic RNA synthesized in virus-infected cells^2^.

All virus-specific enzymatic activities, required for viral RNA synthesis, are present in P1234 and its cleavage products^3^. Allthough uncleaved P1234 possesses several enzymatic activities^4^, it is incapable of performing viral RNA replication at detectable levels^5^. To become active, P1234 must first be processed into the early replicase (P123 polyprotein + nsP4). This complex can, in principle, perform all the essential steps of viral RNA synthesis^6^. However, during alphavirus infection the early replicase is converted into the mature (nsP1 + nsP2 + nsP3 + nsP4) form^7^. All these cleavages are performed by a protease located at the C-terminus of nsP2^8^,^9^,^10^ and the processing of P1234 is regulated at multiple levels^11^,^12^,^13^,^14^.

The protease part of nsP2 can be easily purified as an active recombinant protein^10^,^15^. In addition, the protease activity of nsP2 can be studied using *in vitro* translation and cell culture models^9^,^16^. Early studies showed that alphavirus nsP2 is similar to papaine-like proteases. The catalytic dyad of Sindbis virus (SINV) nsP2 is represented by Cys481 and His558 residues^8^,^17^,^18^; these correspond to Cys478 and His548 in CHIKV and Semliki Forest virus (SFV) nsP2. Mutation of these residues results in the complete loss of protease activity^19^ and abolishes the infectivity of SINV genomes^18^. Different cleavage sites in P1234 are recognized in different ways. Specifically, SFV nsP2 cannot cleave short substrates representing the cleavage site between nsP2 and nsP3 (2/3 site). This cleavage requires both the native N-terminus of nsP2 and a long substrate comprising a few P-side residues followed by approximately 165 N-terminal residues of nsP3^12^,^15^.

The 3D structures of the proteases from Venezuelan equine encephalitis virus (VEEV)^20^, SINV^21^ and CHIKV (Protein Data Bank code 3TRK) reveal that nsP2 protease is a member of MEROPS Clan CN and contains a papain-like protease linked to a C-terminal domain resembling FtsJ-like methyltransferases (MTL domain)^20^. Subsequent molecular modelling revealed the features of the catalytic site and the S1′–S4 subsites^22^. A peptidomimetic inhibitor of nsP2 protease binds at the interface of the protease and MTL domains resulting in conformational change that most probably assists in leaving group departure of either the amine or Cys thiolate during the catalytic cycle^23^; the binding mode of natural substrates is most likely similar. In addition to the catalytic Cys, Asn475 and Lys480 residues (correspond to residues 476 and 481 of CHIKV nsP2) have been shown to be important for the protease activity of VEEV nsP2^23^. Known 3D structure coupled with the functional importance of nsP2 have made this protein an attractive target for the development of inhibitors of alphavirus infection^23^,^24^,^25^,^26^,^27^,^28^.

CHIKV nsP2 possesses all the enzymatic activities known for nsP2 derived from other alphaviruses^29^,^30^. However, data concerning the protease activity of CHIKV nsP2 is conflicting. On one hand, studies performed using the purified protease part of nsP2^31^ or full length nsP2 with a native N-terminus^16^ have demonstrated that CHIKV nsP2 is functionally similar to SFV nsP2. On the other hand, Saisawang and co-authors observed that CHIKV nsP2, derived from East/Central/South African (ECSA) isolate from Thailand, differs from other alphavirus nsP2 proteases in the recognition of small peptide substrates^32^. Recently the same team also reported that the catalytic dyad Cys478 of CHIKV nsP2 could be interchangeable with a proximal Ser482 residue that also contributes to the protease activity of nsP2^33^. Thus, CHIKV nsP2 was reported to have properties very different from those revealed for the ns proteases of other alphaviruses. Indeed, functions of the same ns protein originating from different alphaviruses can be substantially different^34^,^35^. However, in this case the data was obtained using only a single short peptide substrate based assay and the conclusion was not directly confirmed by comparing the enzymes from different alphaviruses in the same experiment. Furthermore, this finding has not been verified using cell-based experiments.

As unusual properties of CHIKV nsP2 would have implications for the molecular biology of this virus as well as for the development of inhibitors of CHIKV infection, we evaluated the properties of CHIKV nsP2 using multiple verified cell-free and cell-based assays. This analysis revealed that Cys478 residue is indispensable for CHIKV ns polyprotein processing, RNA replication and viral infectivity. Substitution of Ser482 residue did not affect any of these crucial functions. Thus, it must be asserted that CHIKV nsP2 is a classical alphavirus ns protease with properties very similar to those previously revealed for SFV nsP2.

## Results

### Substrate requirements for CHIKV nsP2 protease are similar to those of SFV nsP2

**Figure 1:**
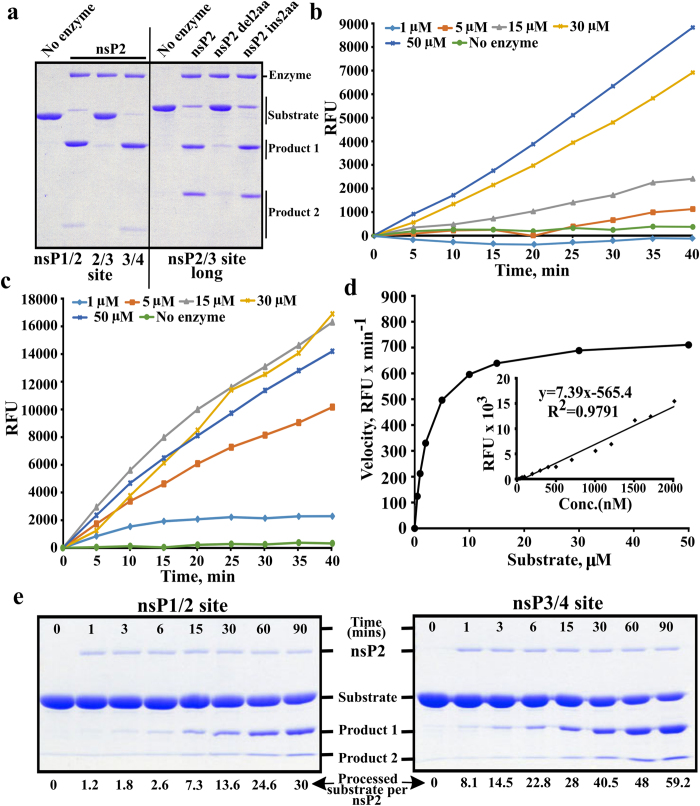
Processing of different substrates by CHIKV nsP2. (**a**) Processing of recombinant protein substrates. Reaction mixtures were incubated for 60 min and reaction products were resolved using 12% SDS-PAGE. Substrates contained either short (10:5) peptides originating from 1/2, 2/3 and 3/4 sites (left side of the panel) or a longer (10:170) region representing 2/3 site and macro domain of nsP3 (right side of the panel). Enzymes used to perform cleavage are shown above the panel. (**b**) Processing of Short peptide substrate. The concentration of nsP2 was kept constant (78 nM) while concentration of the substrate ranged from 1 to 50 μM (shown above graphs); in the control reaction 15 μM of substrate was incubated without the enzyme. The fluorescence resulting from cleavage is shown as the function of time. (**c**) Processing of Long peptide substrate. The experiment was performed as in panel b. (**d**) Enzyme kinetic constant K_m_ was derived by plotting the initial reaction velocity on vertical axis against increasing Long peptide substrate concentration (0.5, 1, 2, 5, 10, 15, 30, 50 μM) on horizontal axis. Inset: representative plot of EDANS fluorescence generated by treating increasing concentrations of Long peptide substrate with proteinase K (50 μg per reaction). (**e**) Processing kinetics of wt nsP2 revealed using recombinant protein based substrates. Wt nsP2 was mixed with substrates corresponding to 1/2 (left panel) and 3/4 (right panel) sites using molar ratio 1:100. Reactions were carried out at 30 °C; aliquots were collected at 0 (before adding enzyme), 1, 3, 6, 15, 30, 60 and 90 min, and analysed using 10% SDS-PAGE. ImageJ program (NIH, USA) was used to quantify substrate band intensities. The values were further analysed using MS Excel software and expressed as pmol of processed substrate per pmol of nsP2 as indicated at the bottom of the figure. All experiments were repeated at least twice; data from one of reproducible experiment is shown.

CHIKV nsP2 readily cleaved GFP-10:5-Trx substrates containing short P10-P5′ sequences originating from the 1/2 and 3/4 sites but not from the 2/3 site. Consistent with the requirement of nsP3 macro-domain for 2/3 site cleavage, the longer substrate GFP-10:170 was efficiently cleaved (Fig. 1a). Another critical determinant for 2/3 site cleavage, revealed in studies of SFV nsP2, is a requirement for the intact native N-terminus of nsP2^12^. It was found that the removal of just two amino acid residues from the N-terminus of CHIKV nsP2 almost completely abolished the processing of GFP-10:170. However, in contrast to SFV nsP2, the addition of two extra amino acid residues (Gly-Ala) to the N-terminus of CHIKV nsP2 had only a minor effect on the cleavage of GFP-10:170 (Fig. 1a).

Next, experiments were performed using fluorogenic peptide substrates. It has been demonstrated that residues P6 to P1 have a clear impact on the cleavage in the context of protein-based substrates and, most importantly, in the context of infectious virus^11^,^15^. Accordingly, peptide substrates used previously have been sub-optimal, as they contained only three or four P-side residues^31^,^32^,^33^. Based on the above considerations, peptide substrates representing the P6-P2′ regions of 1/2 and 3/4 sites were designed. However, due to problems in peptide synthesis, we could not obtain a peptide representing the 1/2 site with required (>90%) purity. Therefore only the substrate with the sequence LDRAGG↓YI (designated as Short), representing the 3/4 site, was used in subsequent experiments. This substrate was processed by CHIKV nsP2, but the reaction velocity could not be saturated (Fig. 1b). This indicated that the Short substrate was still sub-optimal. Previously, we observed that SFV nsP2 cleaves a substrate having 10 amino acid residues from the P-side more efficiently than substrates having only 6 P-side residues (our unpublished data). Therefore a longer substrate, spanning positions P10 to P5′ (DELRLDRAGG↓YIFSS; designated as Long), was used^36^. This substrate was processed more efficiently than Short (compare Fig. 1b,c). Importantly, the reaction velocity reached a plateau (Fig. 1d) indicating that Long peptide represents a preferred tool for functional studies.

The protease domain of SFV nsP2 is capable of hydrolysing a 400-fold molar excess of a protein-based substrate in 5 min indicating a cleavage rate of ≥1.3 s^−1^ or ≥78 min^−1 ^10. This is largely comparable with data observed for many virus-encoded proteases^37^. In contrast, Saisawang and colleagues determined a k_cat_ for CHIKV nsP2 as 0.0016 s^−1^ or 0.096 min^−1 ^33. Using a previously described assay^10^ it was found that in 3 min CHIKV nsP2 was able to process approximately 1.8-fold molar excess of a substrate corresponding to the 1/2 site and approximately 14.5-fold molar excess of a substrate representing the 3/4 site (Fig. 1e). Thus, similar to SFV nsP2, the CHIKV enzyme prefers the 3/4 substrate for cleavage *in trans*. However, in CHIKV the difference in cleavage efficiencies for these two substrates was considerably smaller than previously observed for SFV^10^, probably because both 1/2 and 3/4 sites of CHIKV have preferred P4 Arg residues^15^. The cleavage rate for 3/4 substrate was ≈0.08 s^−1^ or 4.8 min^−1^. This activity is very similar to that observed for VEEV protease domain using similar substrates^23^ and approximately 50-fold higher than reported by Saisawang and colleagues using peptide substrates^33^.

Finally, we performed estimation of K_m_ and k_cat_ using Long substrate and CHIKV nsP2 with a native N-terminus. By carrying out a protease assay with increasing concentration of Long substrate, the apparent K_m_ was found to be 2.5 ± 0.1 μM (Fig. 1d). In order to estimate k_cat_ for this reaction an approach used for SARS coronavirus 3C protease^38^ taking into account secondary quenching of EDANS (5-[(2′-aminoethyl)-amino] naphthalenesulfonic acid) fluorescence, was used. The treatment of Long peptide substrate with 20, 30 or 50 μg of proteinase K reproducibly generated comparable intensity of fluorescence indicating that the substrate was cleaved close to 100% and that proteinase K itself doesn’t affect the fluorescence (Fig. 1d). Using this calibration, the k_cat_ of CHIKV nsP2 for cleavage of Long substrate was found to be ≈2.5 min^−1^, similar to that observed using recombinant protein substrates. Taken together this data also indicates that unlike sub-optimal peptides, a properly designed one (such as Long) does represent a suitable substrate for CHIKV nsP2 protease.

### Substitution of catalytic Cys478 residue blocks the ability of nsP2 to cleave substrates corresponding to 1/2 and 3/4 sites

**Figure 2:**
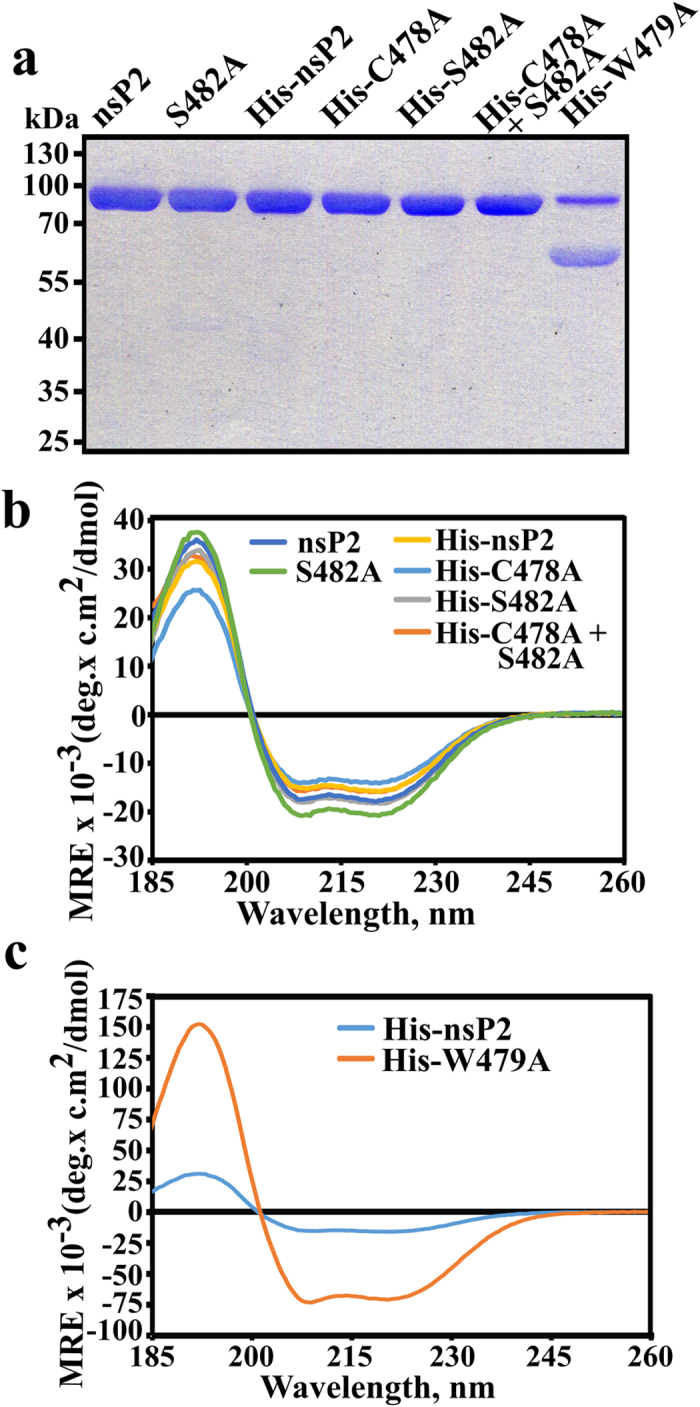
Purification of recombinant nsP2 and its mutant forms. (**a**) SDS-PAGE analysis of purified recombinant enzymes. The proteins are shown above the panel; each lane corresponds to approximately 1 μg of purified protein. (**b**) CD spectroscopy analysis of the nsP2, nsP2^S482A^, His-nsP2, His-nsP2^C478A^; His-nsP2^S482A^ and His-nsP2^C478A+S482A^. All of these recombinant proteins were found to be properly and comparably folded. (**c**) Comparison of CD spectra of His-nsP2 and His-nsP2^W479A^. The shapes of the spectra are identical (compare also with panel b) but His-nsP2^W479A^ produces a strongly enhanced signal.

We mutated the proposed catalytic Cys478 residue of CHIKV nsP2 to Ala (C478A), the Ser482 residue, reported to be able to substitute for Cys478 residue^33^ to Ala (S482A), and combined these mutations (C478A + S482A). We also mutated the Trp479 residue, adjacent to catalytic Cys478, to Ala (W479A). To obtain purified mutant nsP2 proteins, we first took advantage of the ability of CHIKV nsP2 to tolerate N-terminal tags. Using this approach recombinant wild type (wt) His-nsP2, His-nsP2^C478A^, His-nsP2^S482A^ and His-nsP2^C478A+S482A^ were all obtained at high purity (Fig. 2a). The identities of all of recombinant proteins were confirmed using mass-spectrometry. The purification of His-nsP2^W479A^ turned out to be difficult; the protein contained a substantial amount of a faster migrating contaminating protein (Fig. 2a). No major *E. coli* derived protein was revealed by mass-spectrometry in His-nsP2^W479A^ indicating that the faster migrating protein represents truncated form(s) of His-nsP2^W479A^. Next, the CD-spectra of all purified recombinant proteins were obtained and compared to each other. This analysis revealed that all these proteins were folded similarly with no detectable structural deformity from nsP2 or His-nsP2 (Fig. 2b,c). However, His-nsP2^W479A^ exhibited a CD spectrum with abnormally high ellipticity (Fig. 2c). Together with altered size-exclusion chromatography profiles (not shown) this indicates that His-nsP2^W479A^ most probably forms high molecular weight aggregates.

**Figure 3:**
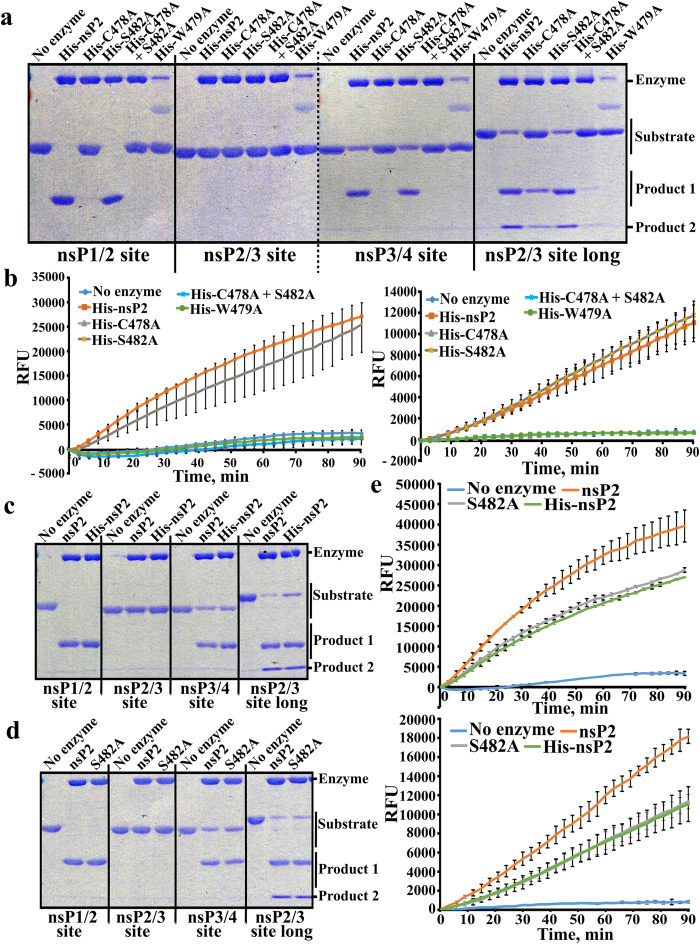
Effects of C478A, W479A and S482A substitutions on the protease activity of CHIKV nsP2. (**a**) Comparison of protease activities of His-nsP2, His-nsP2^C478A^, His-nsP2^S482A^, His-nsP2^W479A^ and His-nsP2^C478A+S482A^ using recombinant protein as substrates. The assay was performed and results analysed as described for Fig. 1a. The dotted vertical line on panel indicates merging of two different gels. (**b**) Comparison of protease activities of His-nsP2, His-nsP2^C478A^, His-nsP2^S482A^, His-nsP2^W479A^ and His-nsP2^C478A+S482A^ using the continuous FRET based assay. 1 μg of His tagged nsP2 or its mutants was mixed with 15 μM Long (left panel) or Short (right panel) substrate in the final reaction volume of 200 μL/well. The 96-well plate was placed in the Synergy MX reader and the reaction was carried out at 30 °C for 90 min. Recorded EDANS fluorescence (RFU, relative fluorescence units) is shown at the vertical axes. (**c**) Comparison of wt nsP2 and His-nsP2 using recombinant protein based substrates. Assays were performed and data is presented as described for Fig. 1a. (**d**) Comparison of wt nsP2 and nsP2^S482^ using recombinant protein based substrates. Assays were performed and data is presented as described for Fig. 1a. (**e**) Comparison of wt nsP2, nsP2^S482^ and His-nsP2 using Long (upper panel) and Short (lower panel) peptide substrates. Assays were performed and data is presented as described for panel **b**. Experiments were repeated at least twice; data from one of reproducible experiment is shown.

Next we compared the abilities of His-tagged nsP2 proteins to cleave recombinant protein-based substrates. None of the enzymes was able to cleave recombinant protein containing a short version of the 2/3 site with detectable efficiency. His-nsP2^S482A^ was, however, capable of cleaving substrates containing 1/2, 3/4 and 2/3 (long) sites with efficiency similar to that of wt His-nsP2 (Fig. 3a). His-nsP2^W479A^ was completely inactive, with the caveat of formation of inactive aggregates. Importantly, His-nsP2^C478A^ and His-nsP2^C478A+S482A^ were both also completely unable to cleave 1/2 and 3/4 substrates (Fig. 3a). Surprisingly, both of these mutants were able to cleave, albeit with much reduced efficiencies (compared to wt His-nsP2), 2/3 (long) substrate (Fig. 3a). This result highlights the unique nature of 2/3 site cleavage. Peptide substrates led to results that were identical to those obtained for the corresponding (3/4) recombinant protein based substrate: His-nsP2^C478A^, His-nsP2^C478A+S482A^ and His-nsP2^W479A^ were all defective in cleavage of both Long and Short substrates while His-nsP2 and His-nsP2^S482A^ had similar activities (Fig. 3b).

As His-tag alters several enzymatic and non-enzymatic properties of nsP2^29^ it was important to compare the activities of His-tagged nsP2 with those of non-tagged protein. Direct comparison of wt nsP2 and His-nsP2 confirmed that both proteins are active. Using recombinant protein substrates, we were unable to detect significant differences between these two enzymes (Fig. 3c). The assay using peptide substrates revealed that His-nsP2 was slightly (<2-fold) but reproducibly less active than wt nsP2 (Fig. 3e). Thus, the N-terminal His-tag has a small influence on protease activity of CHIKV nsP2.

Next, we introduced the S482A mutation into pET-nsP2 vector designed for production of nsP2 with authentic N-terminus *via* removal of the N-terminal tag by the protease activity of the protein itself^29^. NsP2^S482A^ was successfully purified (Fig. 2a). This approach was not applicable for other mutant nsP2 proteins, which were clearly unable to process 1/2 and 3/4 substrates (Fig. 3a,b). Comparison of nsP2 and nsP2^S482A^ in the same experiment revealed no differences in the cleavage of recombinant protein based substrates. Expectedly, both enzymes were also capable of cleaving peptide substrates; in these assays nsP2^S482A^ was slightly, but reproducibly, less active than wt nsP2 (Fig. 3e).

### Cys478 and Trp479 residues of nsP2 are required for processing of CHIKV P1234 polyprotein

Our analysis carried out using recombinant proteins and different types of substrates shows that unlike reported recently^33^, Cys478 residue is indispensable and irreplaceable for the ability of CHIKV nsP2 to process any substrate corresponding to 1/2 and 3/4 sites. However, the used systems are artificial: in alphavirus infection the polyprotein context may affect both the activity of the enzyme and the presentation of its substrates^11^.

**Figure 4:**
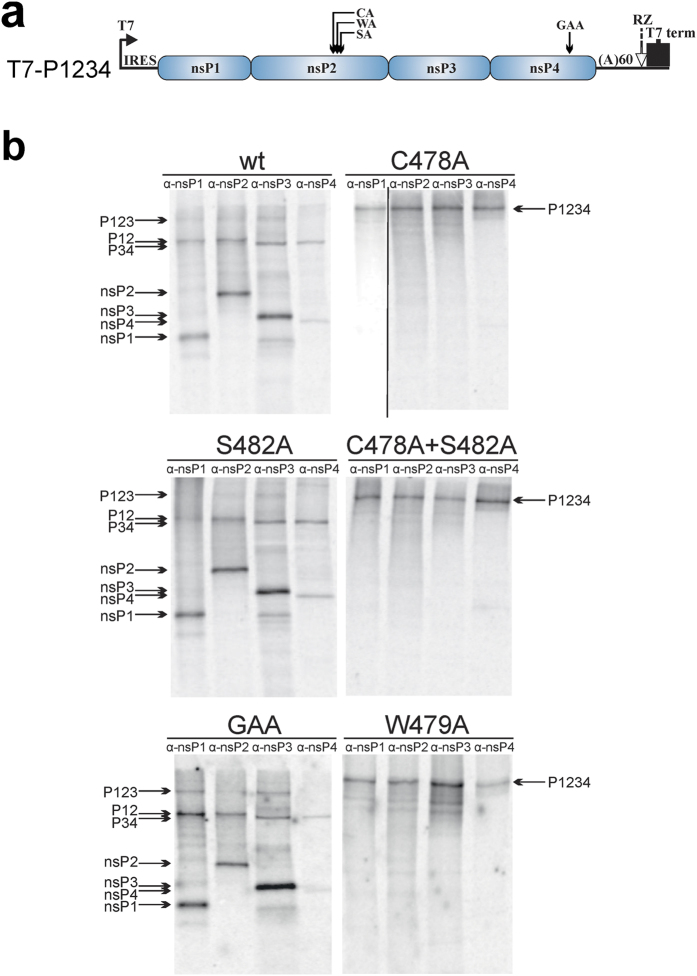
C478A and W479A substitutions in nsP2 region block the processing of CHIKV P1234 in cell-free system. (**a**) Schematic presentation of the T7-P1234 plasmid used for the assay; mutations in nsP2 or nsP4 present in T7-P12^CA^34, T7-P12^WA^34, T7-P12^SA^34, T7-P12^CA+SA^34 and T7-P1234^GAA^ are indicted above the drawing. IRES–internal ribosome entry site of encephalomyocarditis virus; RZ–antigenomic strand ribozyme from hepatitis D virus. (**b**) 2 μg of a T7-P1234, T7-P12^CA^34, T7-P12^WA^34, T7-P12^SA^34, T7-P12^CA+SA^34 or T7-P1234^GAA^ plasmids were used as templates for *in vitro* transcription and translation carried out in the presence of 20 μCi of [^35^S]methionine-cysteine mixture. Reactions were stopped by adding cycloheximide, CHIKV ns proteins and polyproteins were immunoprecipitated using antisera against nsP1, nsP2, nsP3 and nsP4. The precipitated proteins were separated by 8% SDS-PAGE and visualized using a Typhoon imager. Mutations introduced into ns polyproteins are shown above the panels, antibodies used for immunoprecipitation are indicated at the top of the respective lanes. Positions of ns proteins and polyproteins are indicated by arrows. The vertical line on C478A panel indicates merging of images obtained by different exposure, for full images see Supplementary Information. The experiment was repeated twice, data from one of reproducible experiment is shown.

To analyse the effects of mutations in the nsP2 active site region on the processing of CHIKV P1234 polyprotein C478A, W479A, S482A and C478A + S482A substitutions were introduced into T7-P1234 plasmid (Fig. 4a)^39^. The plasmids, together with wt T7-P1234 and T7-P1234^GAA^ polymerase mutant controls^39^, were transcribed/translated *in vitro,* CHIKV ns proteins were precipitated using antibodies against nsP1, nsP2, nsP3 and nsP4 and resolved by SDS-PAGE. The only specific translation product of T7-P12^CA^34, T7-P12^WA^34 and T7-P12^CA+SA^34 was a large polyprotein; precipitation by all four antibodies confirmed that it was P1234 (Fig. 4b). We could not reliably detect products that could correspond to P12 and P34 polyproteins that would result from the residual processing of 2/3 site by mutant proteases. Thus, it should be concluded that protease present in P12^CA^34, P12^WA^34 and P12^CA+SA^34 polyproteins were completely inactivated. This data confirms that Ser482 cannot substitute for Cys478. The lack of processing of T7-P12^WA^34 encoded polyprotein indicates that Trp479 is also crucial for nsP2 protease activity. In contrast, all mature ns proteins were detected among the products obtained from T7-P1234, T7-P12^SA^34 and T7-P1234^GAA^ (Fig. 4b). The amounts of P1234 polyprotein were typically too low to be detected; however, several processing intermediates (P123, P12, P34) were clearly identified (Fig. 4b). Thus, the S482A substitution had no detectable effect on P1234 processing.

### C478A and W479A but not S482A substitution block processing of P1234 in transfected cells and inhibit CHIKV *trans-*replicase

**Figure 5:**
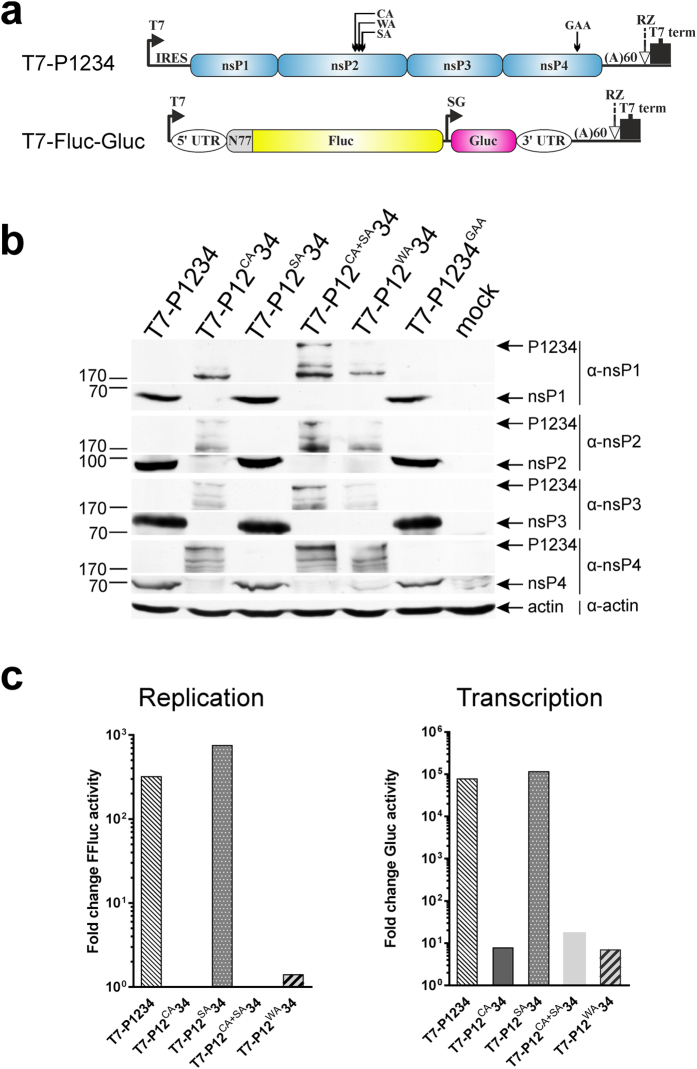
C478A and W479A substitutions in nsP2 region abolish processing of P1234 and CHIKV *trans-*replication system mediated activation of reporter expression. (**a**) Schematic presentation of T7-P1234 plasmid encoding for CHIKV replicase and T7-Fluc-Gluc plasmid encoding for replication competent template RNA carrying sequences encoding for Fluc and Gluc reporter proteins. Mutations introduced into nsP2 or nsP4 are shown as in Fig. 4a. IRES – internal ribosome entry site of encephalomyocarditis virus; RZ–antigenomic strand ribozyme from hepatitis D virus, UTR–untranslated region from CHIKV genome, SG–CHIKV subgenomic promoter spanning residues −78 to +69 with respect to the start position of subgenomic RNA, N77–region encoding for the 77 N-terminal amino acid residues of nsP1. (**b**) BSR cells were transfected with T7-P1234, T7-P12^CA^34, T7-P12^WA^34, T7-P12^SA^34, T7-P12^CA+SA^34 or T7-P1234^GAA^. At 18 h p.t. cells were lysed with Laemmli buffer, proteins were separated by 8% SDS-PAGE, transferred to membrane and detected using Western blotting and antibodies against CHIKV nsP1, nsP2, nsP3 and nsP4. Parts of the same gels corresponding to mature ns proteins and P1234 polyproteins are shown, as due to lower efficiency of detection P1234 and products of its degradation cannot be revealed using the same exposure used for detection of mature ns proteins; for full-length images see Supplementary Information. Positions of ns proteins and P1234 polyprotein are indicated by arrows on the right, positions of molecular mass markers (in kDa) are shown on the left. Actin was used as loading control. (**c**) BSR cells were co-transfected with T7-P1234, T7-P12^CA^34, T7-P12^WA^34, T7-P12^SA^34, T7-P12^CA+SA^34 (indicated below the graph) or T7-P1234^GAA^ (control) and T7-Fluc-Gluc. At 18 h p.t. cells were lysed, activities of Fluc and Gluc were measured and normalized to those measured for control cells. Normalized activities of Fluc were used to estimate synthesis of full-length template RNA (Replication) by CHIKV *trans-*replicase; normalized Gluc activities were used to estimate synthesis of subgenomic RNA (Transcription). Data from one out of three reproducible independent experiments is shown.

Mutations that have a strong effect on the processing of CHIKV P1234 in *in vitro* transcription/translation system do not always have the same effect in living cells^16^,^39^. Therefore BSR T7/5 (hereafter BSR) cells, a derivative of BHK-21 cells stably expressing T7 RNA polymerase^40^, were transfected with wt polyprotein T7-P1234 expression construct and its mutant derivatives (Fig. 5a). Cells were lysed at 18 h post transfection (h p.t.) and ns polyproteins and their processing products were analysed using Western blotting. A full set of mature ns proteins was detected in cells transfected with T7-P1234, T7-P12^SA^34 or T7-P1234^GAA^ (Fig. 5b); coherent with previous observations^39^ no ns polyproteins could be detected. In contrast, large P1234 polyprotein was the only distinct product detected in T7-P12^CA^34, T7-P12^WA^34 or T7-P12^CA+SA^34 transfected cells; no mature ns proteins or polyproteins that could be identified as P12 or P34 were observed (Fig. 5b). Thus, effects of C478A, W479A and S482A mutations on CHIKV ns polyprotein processing observed in cell-free assays were also reproduced in cell-based assay.

The functional importance of any mutation in the alphavirus replicase is best assessed by the ability of the viral replicase to perform the amplification and transcription of suitable RNA templates. Here we took advantage of a recently developed CHIKV *trans-*replicase system^39^. BSR cells were co-transfected with the T7-Fluc-Gluc plasmid (Fig. 5a) producing a reporter-containing replication-competent RNA^39^ and plasmids expressing different polyprotein variants. Expression of the firefly luciferase (Fluc) marker, encoded by the 5′ ORF of the replicating RNA template, was amplified more than 300-fold in the presence of T7-P1234 or T7-P12^SA^34 (Fig. 5c, left panel). In contrast, no amplification of Fluc expression was observed in the presence of T7-P12^CA^34, T7-P12^WA^34 or T7-P12^CA+SA^34 (Fig. 5c, left panel).

The *Gaussia* luciferase (Gluc) reporter is translated exclusively from replicase-generated subgenomic RNAs; thus its expression is strictly dependent on the ability of the replicase to synthesize RNA^39^. Compared to the inactive polymerase mutant, wt replicase activated Gluc expression nearly 100,000-fold. Similar activation was observed for T7-P12^SA^34 (Fig. 5c, right panel) confirming that fully functional replicase complexes were assembled. Somewhat surprisingly, extremely low, but still detectable (≈10-fold) activation of Gluc expression was also observed in the presence of constructs harbouring C478A, W479A or C478A + S482A substitutions (Fig. 5c, right panel). The reason(s) for this small activity remain currently unknown though it is possible that unprocessed P1234 may possess minimal RNA synthesis ability. As these effects are extremely minor (activation of Gluc expression is reduced ≈10,000-fold compared to wt P1234), they were only noticed using extremely sensitive CHIKV *trans*-replicase reporter assay. It is likely that this activity is too low to have biological significance.

### C478A and W479A mutations in P1234 polyprotein abolish RNA synthesis by CHIKV and SFV replicases

**Figure 6:**
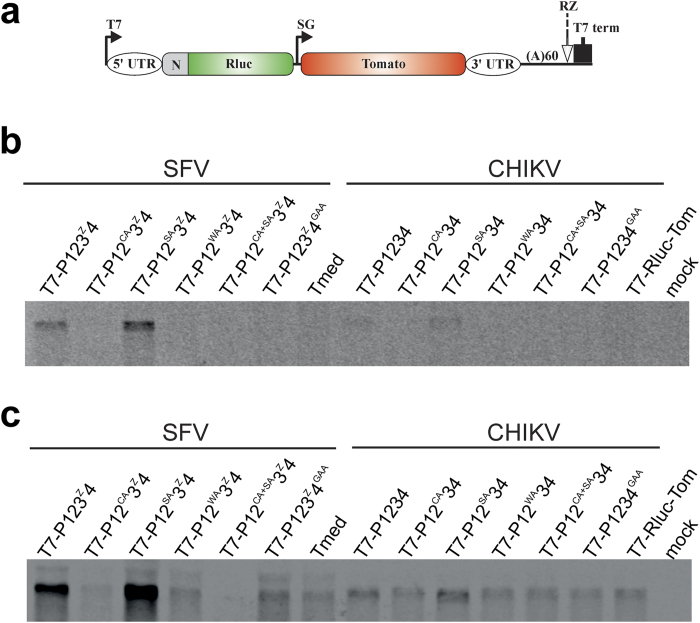
RNA synthesis of CHIKV and SFV *trans*-replicases. BSR cells were co-transfected with plasmid encoding for wt or mutant CHIKV replicase and T7-Rluc-Tom encoding template for CHIKV replicase. Alternatively, cells were transfected with plasmids expressing SFV replicase and Tmed encoding for SFV specific template. Total RNA was isolated at 18 h p.t. Eight micrograms total RNA was fractionated and transferred to Hybond-N+ nylon filter. Negative- and positive-strand RNAs were detected by hybridization with probes targeting the Rluc gene present in the template constructs. (**a**) Schematic presentation of T7-Rluc-Tom and Tmed plasmids encoding for replication competent template RNA of CHIKV and SFV. Plasmids have similar design except that number of codons from ORF1 of corresponding virus (designated as N) is 77 for T7-Rluc-Tom and 74 for Tmed. Other designations are the same as used for Fig. 5a. (**b**) Synthesis of negative-strand RNAs by SFV and CHIKV *trans*-replicases. (**c**) Synthesis of positive-strand full-length RNAs by SFV and CHIKV *trans*-replicases. Parts of the blots corresponding to template RNA are shown on panels b and c; for full-length images see Supplementary Information. The experiment was repeated trice with similar results.

Unlike positive-strand RNA, synthesis of negative-strand RNA cannot be revealed using reporter proteins^41^. Therefore the activity of CHIKV replicases harbouring nsP2 mutations was also analysed using Northern blotting. SFV replicase, harbouring the same set of mutations, was used for comparison. For unknown reasons, CHIKV replicase was rather inefficient in using template RNA derived from T7-Rluc-Tom vector (Fig. 6a); in contrast SFV replicase efficiently utilized template RNA derived from Tmed^42^. Nevertheless, it was clearly and consistently detected that in addition to wt replicases, only replicases harbouring S482A substitution were capable of negative-strand RNA synthesis (Fig. 6b). In fact, the synthesis of negative strands was slightly but reproducibly increased by this mutation, most likely due to slight reduction of protease activity of nsP2 (Fig. 3e) leading to increased half-life of early replicase complex.

It was also found that levels of positive-strand RNAs exceeded background levels (detected in the absence of enzymatically active replicase) only in the presence of wt replicases and those harbouring S482A substitution (Fig. 6c). Together with data obtained for negative-stand RNA synthesis, this confirms that only these replicases are active. This data complements our previous findings and clearly indicates that replicases harbouring C478A, W479A or C478A + S482A substitutions are incapable of both negative- and positive-strand RNA synthesis. Importantly, the activities of SFV replicase were affected by all protease active site mutations in the same way as those of CHIKV replicase (Fig. 6b,c). This is consistent with our data highlighting that the properties of CHIKV nsP2 protease are very similar to those previously revealed for SFV nsP2^10^,^12^,^14^,^15^.

### C478A and W479A but not S482A substitutions block the rescue of infectious CHIKV

**Figure 7:**
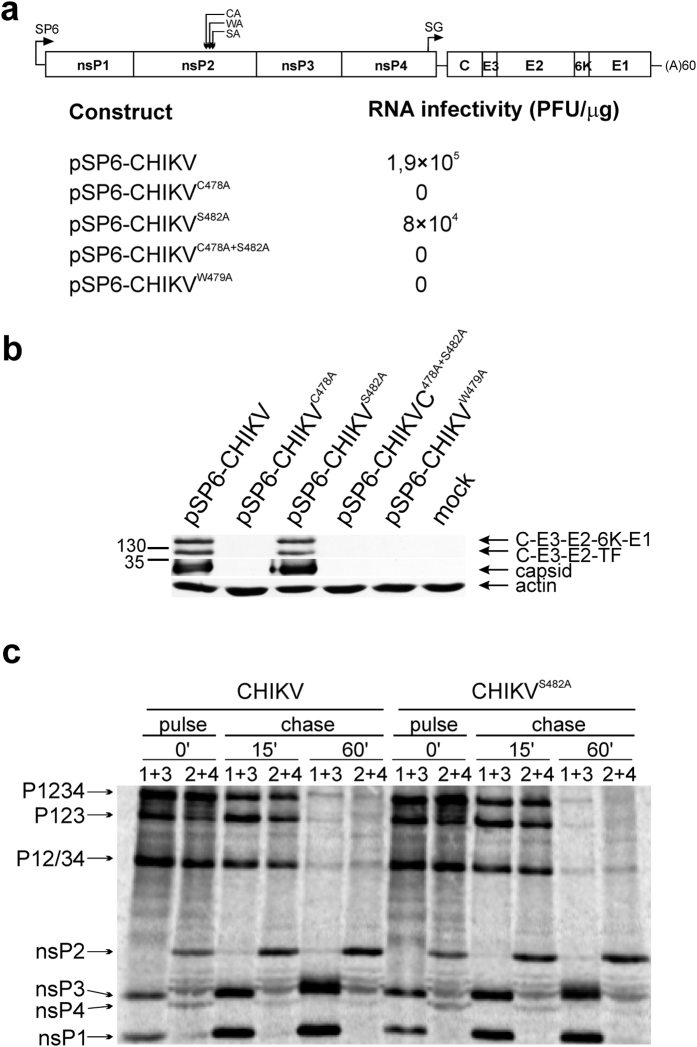
S482A substitution in nsP2 region does not interfere with the rescue of CHIKV or with processing of ns polyproteins in infected cells. (**a**) Schematic presentation of CHIKV genomes (SP6–SP6 RNA polymerase promoter present in the cDNA clone) and results of infectious centre assay. (**b**) BHK-21 cells were transfected with 10 μg of *in vitro* synthesized capped transcripts and lysed either at 18 h p.t. (pSP6-CHIKV and pSP6-CHIKV^S482A^ transcripts) or 144 h p.t. (pSP6-CHIKV^C478A^, pSP6-CHIKV^W479A^ or pSP6-CHIKV^C478A+S482A^ transcripts). Proteins were separated using 10% SDS-PAGE, transferred to membrane and capsid protein was detected using a polyclonal antiserum. Parts of the same gels corresponding to capsid protein and C-E3-E2-6K-E1/C-E3-E2-TF polyproteins are shown; due to lower efficiency of detection, polyproteins cannot be revealed using the same exposure as used for the detection of mature capsid protein; for full-length images see Supplementary Information. Arrows on the right indicate positions of detected proteins; positions of molecular mass markers (in kDa) are shown on the left. Actin was used as loading control. (**c**) BHK-21 cells were infected with wt CHIKV or CHIKV^S482A^ at MOI 20. 3 h post-infection cells were starved in methionine-cysteine-free DMEM (Life Technologies) and labelled with 50 μCi of [^35^S]methionine-cysteine mixture (Perkin-Elmer) for 15 min (pulse, P). In the chased (C) samples the pulse was followed by a chase for 15 or 60 min. Ns proteins were immunoprecipitated using combinations of antibodies: against nsP1 and nsP3 or against nsP2 and nsP4. Immunoprecipitated proteins were separated using 8% SDS-PAGE and visualized using a Typhoon imager. Positions of ns proteins and ns polyproteins are indicted by arrows.

We verified the effects of the protease mutations also in the context of CHIKV RNAs obtained by transcription of infectious cDNA clones (Fig. 7a). Infectious centre assay (ICA) performed after RNA transfection revealed that the rescue efficiencies of wt CHIKV and CHIKV^S482A^ were similar (Fig. 7a). No plaques were detected for cells transfected with the three other mutants. Next, cells were transfected with 10 μg RNA transcripts (corresponding to ≈10^6^ plaque forming units (PFU) for wt CHIKV). Cells transfected with wt CHIKV and CHIKV^S482A^ transcripts displayed strong cytotoxic effects by 18 h p.t. In contrast, no cytotoxicity was observed for the other transcripts even at 144 h p.t. To study the presence of replicating virus genomes in the cells, all the transfected cultures were analysed for the presence of capsid protein (expression of which is completely dependent on viral RNA replication). High amounts of mature capsid protein (as well as some amounts of its precursors) were detected in cells transfected with transcripts of pSP6-CHIKV and pSP6-CHIKV^S482A^. In contrast, no capsid protein could be detected in cells transfected with pSP6-CHIKV^C478A^, pSP6-CHIKV^W479A^ or pSP6-CHIKV^C478A+S482A^ transcripts (Fig. 7b). Furthermore, while wt CHIKV and CHIKV^S482A^ reached comparable high titers (7.5 × 10^8^ and 6.5 × 10^8^ PFU/mL, respectively) and formed plaques of similar size (not shown) no infectious virus was detected in the other three samples. Taken together, this data unequivocally demonstrates that mutations C478A and W479A not only abolish ns polyprotein processing but are also lethal for CHIKV.

To analyse the effect of S482A mutation on ns polyprotein processing in the context of CHIKV infection, a pulse-chase experiment was performed. For wt CHIKV infected cells all ns proteins, P1234 precursor and P123, P12 and/or P34 processing intermediates were detected in pulsed samples (Fig. 7c). In samples chased for 15 min the amounts of P1234 were reduced; after 60 min chase all ns polyproteins were poorly detectable. In contrast, the amounts of mature ns proteins (with the exception of unstable nsP4) increased during the chase. No significant differences from this pattern were observed for CHIKV^S482A^ (Fig. 7c). This data confirms that S482A substitution has no negative effect on ns polyprotein processing in CHIKV infected cells.

**Figure 8:**
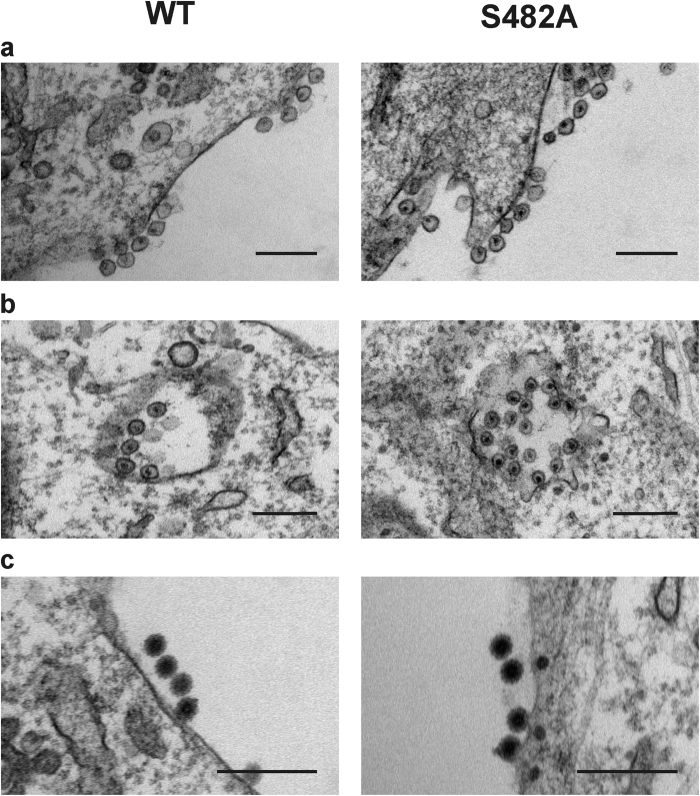
CHIKV^S482A^ forms spherules, CPVs and virions similar to wt CHIKV. BHK-21 cells were infected with wt CHIKV and CHIKV^S482A^ at MOI 10. At 8 h post-infection samples were fixed and processed for transmission electron microscopy. Scale bar corresponds to 200 nm. (**a**) Spherules (replication complexes) formed on the plasma membrane of infected cells. (**b**) Internalized CPVs containing multiple spherules. (**c**) Progeny virions budding out from the plasma membrane.

Finally, it was analysed whether the S482A substitution affects the replication complex (spherule), replication organelle (cytopathic vacuoles, CPV) or progeny virion formation. Comparison of wt CHIKV and CHIKV^S482A^ revealed no detectable differences in the formation of any of these virus-specific structures (Fig. 8) confirming that Ser482 residue is dispensable for the CHIKV infectious cycle.

## Discussion

Thus far the data regarding the properties of CHIKV nsP2 protease has been controversial. As this enzyme is central for CHIKV infection and represents a favoured target for anti-CHIKV inhibitor development, revealing the processing requirements of the enzyme and the significance of residues located in its active site region was essential.

The easiest way to study protease activities is to use a purified enzyme and corresponding substrate(s). However, as during the infection process the enzymatic activities of viral enzymes are affected by multiple factors, using a single type of assay provides poor grounds to draw conclusions about the properties of nsP2, including quantitative efficacy. Furthermore, cell-free assay does not provide information about the biological relevance of the observed effects.

Here it was observed that the substrate requirements of CHIKV nsP2 protease were similar to those reported for its SFV counterpart. At the same time the protease activity of CHIKV nsP2 was considerably lower than that previously reported for the protease domain of SFV nsP2^10^. Most likely this reflects differences in the basic enzymatic activities of the proteases from different alphaviruses. CHIKV nsP2 harbours a Glu residue in position 515 that is predicted to be part of the S4 subsite of the enzyme^22^. In contrast, the SFV protease has Val515 that was shown to increase the ability of nsP2 to process P34 polyprotein in infected cells^43^. Thus, it is possible that in order to compensate for the presence of preferred P4 Arg residue in the 1/2 site of P1234 polyprotein, CHIKV nsP2 must have lower basal protease activity; the same has previously been observed for the avirulent A7(74) strain of SFV^43^.

The current work also reinforces the crucial role of Cys478 residue in the catalytic site of nsP2 protease. We found no evidence that the enzyme harbouring C478A mutation is capable of cleaving any versions of substrates corresponding to 1/2 and 3/4 sites. Furthermore, this mutation blocked all the essential biological activities of the corresponding replicases and virus. Expectedly, no detectable cleavage of short 2/3 substrate by any recombinant enzyme was observed. However, with the exception of His-nsP2^W479A^ that likely forms enzymatically inactive aggregates, all the used enzymes were capable of inducing the cleavage of 2/3 long substrate. Again, Cys478 but not Ser482 residue was crucial for full efficiency of that cleavage reaction; however, when Cys478 was substituted with Ala, low level processing was still observed. The mechanism responsible for this process remains enigmatic though it certainly was not due to the proposed compensatory effect of Ser482 residue (Fig. 3a). Biophysical or perhaps even structural experiments are required to clarify mechanism(s) responsible for this cleavage. Interestingly, it has been previously observed that processing of 2/3 long substrate is also insensitive to the effect of Pro718 to Gly mutation in nsP2 that severely reduces processing of other substrates^16^. This indicates that due to its unique macromolecular assembly dependent mode of cleavage, the processing of 2/3 site is greatly enhanced by, but not totally dependent on, the Cys478 residue. It should, however, be emphasized that the unusual processing of 2/3 site was not detected upon more natural conditions, such as processing of P1234 in the test tube or in cells and that Cys478 to Ala mutation was irreparably lethal. Thus, it is possible that this residual cleavage activity may represent an artefact of cell-free assays.

The sequence of the active site motif of CHIKV nsP2 (476-NVCWAKS-482) is very similar to that of VEEV nsP2 (475-NVCWAKA-481). Interestingly, the significance of residues 475 and 480 of nsP2 of VEEV (corresponding to Asn476 and Lys481 in CHIKV nsP2) for protease activity has been recently demonstrated. The substitution of Asn residue with Ala led to 25-fold reduction in k_cat_/K_m_ of the enzyme. Mutation of Lys residue to Ala led to a 9-fold decrease in k_cat_ and k_cat_/K_m_^23^. Thus, together with the data presented in this study, the importance of most residues in the active site motif of alphavirus nsP2 protease has been demonstrated. Two conserved residues (Cys478 and Trp479) are critically important for protease activity; residues N476 and K481 modulate the activity of the enzyme while substitution of Ser482 to Ala, a residue naturally occurring in the analogous position of VEEV and many other alphaviruses, has little to no effect. For different alphaviruses corresponding position can be occupied also by Cys or Thr residues indicating that considerable variation is tolerated in this position.

Alphavirus nsP2 proteases have not been systematically compared to each other using identical experimental conditions. However, similarities in 3D structures and data from the analysis of functionally important residues strongly indicate that there are no significant differences, at least regarding the functional significance of active site motif residues, between the enzymes encoded by different alphaviruses. CHIKV nsP2 is not an exception; it is a typical alphavirus cysteine protease. Contrary to the recent report, the Ser482 residue from the active site motif of CHIKV nsP2 cannot to any extent compensate for the loss of the catalytic Cys478 residue. The conserved nature of CHIKV nsP2 with its counterparts from different alphaviruses indicates that similar approaches can be used to study these enzymes and their roles in virus infection as well as develop and select for inhibitors of different alphavirus proteases. This opens the possibility that one compound could inhibit nsP2 proteases from different alphaviruses.

## Methods

### Cells and media

All cells were grown at 37 °C in 5% CO_2_ atmosphere; 10% fetal bovine serum, 100 U/mL penicillin and 0.1 mg/mL streptomycin. BHK-21 cells (ATCC CCL-10) were grown in Glasgow’s Minimal Essential Medium (GMEM, Gibco) containing 2% tryptose phosphate broth and 200 mM HEPES. BSR cells were cultured in the same medium supplemented with 1 mg/mL of G418.

### Construction of plasmids for expression of mutant forms of CHIKV nsP2

Plasmids pET-nsP2, designed for expression of CHIKV nsP2 (ECSA genotype, isolate LR-2006-OPY1) with native N-terminus, and pET-His-nsP2, encoding CHIKV nsP2 preceded by amino acid sequence MNHHHHHH-SGGGS-ENLYFQ^29^, were used for expression of wt nsP2 and wt His-nsP2 proteins, respectively. Expression vectors designated pET-nsP2^S482A^, pET-His-nsP2^C478A^, pET-His-nsP2^C478A+S482A^, pET-His-nsP2^W479A^ and pET-His-nsP2^S482A^ containing mutations resulting in the specified amino acid substitutions in nsP2 were generated *via* PCR-based site-directed mutagenesis. Plasmids pET-nsP2-delN2 and pET-nsP2-insN2^29^ were used for expression of nsP2 with deletion of 1^st^ and 2^nd^ amino acid residues at the N-terminus or with addition of GA dipeptide to the N-terminus, respectively. The sequences of all the expression constructs were verified through DNA sequencing.

### Expression and purification of recombinant proteins

EGFP-10:5-Trx substrates, containing short versions of the 1/2, 2/3 or 3/4 sites, as well as the longer version of the substrate corresponding to 2/3 site (EGFP-10:170) were purified as previously described^16^. The expression and purification of wt His-nsP2, His-nsP2^C478A^, His-nsP2^C478A+S482A^ and His-nsP2^S482A^ was performed as previously described^29^. Due to very low expression level purification of His-nsP2^W479A^ protein was performed from 3 litres of induced *E. coli* culture. After loading the cleared cell lysate onto the Ni-affinity column an additional washing step with buffer containing 5 mM ATP:Mg and *E. coli* denatured proteins (0.1 mg/mL) was performed in order to remove the contaminating proteins^44^. It was also observed that His-nsP2^W479A^ did not properly bind to the cation exchange column and remained mostly in the column flow-through, which was therefore used for its further purification using procedures applied for other His-nsP2 proteins. From Superdex 200 10/300GL size-exclusion column (GE Healthcare, UK) His-nsP2^W479A^ eluted with much shorter retention time compared to other nsP2 proteins. Proteins wt nsP2, nsP2-delN2 and nsP2-insN2 were purified using previously described self-cleavage based procedure^29^; the same approach was used to obtain purified nsP2^S482A^. Protein concentrations were measured using Nanodrop spectrophotometer (Thermo Scientific, USA); proteins were flash-frozen and stored at −80 °C. The authenticity of all the purified proteins was verified by mass spectrometric analysis.

### CD spectroscopy

Circular dichroism (CD) spectrum measurements for recombinant wt nsP2, nsP2^S482A^, His-nsP2, His-nsP2^C478A^, His-nsP2^W479A^, His-nsP2^S482A^ and His-nsP2^C478A+S482A^ were carried out as previously described^16^. Briefly, CD spectra were recorded on a Chirascan plus CD spectrometer (Applied Photophysics) three consecutive times in the far-UV region from 185 to 260 nm at a protein concentration of 0.8 μM (with the exception of His-nsP2^W479A^ which was at 0.5 μM) in CD buffer (10 mM KH_2_PO_4_, pH 7.2 and 100 mM NaF). The final spectra were obtained after deducting the mean spectra of buffer from the mean spectra of protein samples.

### nsP2 protease assays

A protease assay using recombinant proteins as substrates was performed as previously described^16^. Reaction mixtures were incubated at 30 °C for 60 min, followed by the addition of 2xSDS loading buffer. Samples were boiled and resolved using 10% SDS-PAGE. To analyse processing kinetics, wt nsP2 was mixed with recombinant proteins containing cleavage sequences (10:5) from 1/2 and 3/4 sites using molar ratio 1:100. The reaction was carried out in 150 μL volume using previously described^16^ conditions; 15 μL aliquots were collected at 0 (before adding enzyme), 1, 3, 6, 15, 30, 60 and 90 min and analysed using 10% SDS-PAGE.

Continuous protease assays were performed using a fluorescence resonance energy transfer (FRET)-based approach. Two types of peptide substrates were used: peptide comprising 15 amino acid residues (DELRLDRAGG↓YIFSS) or 8 amino acid residues (LDRAGG↓YI) from the 3/4 site (arrow indicates the scissile bond). These substrates, designated respectively as Long and Short, were purchased from GenScript (USA). Both substrates contained 4-[[4-(dimethylamino)phenyl]-azo] benzoic acid (DABCYL) and EDANS at the amino and carboxy termini, respectively. The fluorogenic reactions were performed as previously described^36^.

### Determination of Km and Kcat for wt nsP2 of CHIKV using FRET assay

Michaelis-Menten plot (Fig. 1d) was generated by performing nsP2 protease reaction at various concentrations (0.5, 1, 2, 5, 10, 15, 30, 50 μM) of substrate. The velocity of the reaction was linear over several minutes indicating that the enzyme was working at its maximum velocity. Hyperbolic plot was generated by plotting initial velocity against the increasing substrate concentration.

EDANS standard curve was generated after considering the inner filter effect^45^. Briefly, increasing concentration of substrate (0 to 2000 nM as shown in Fig. 1d inset) were treated with 20, 30 or 50 μg of proteinase K (Thermo Scientific) in a final reaction volume of 200 μL at 37 °C for 1 h. The relative fluorescence units (RFU) of EDANS were measured on a Synergy MX microplate reader (BioTek, USA) at excitation wavelength of 340 nm and emission wavelength of 490 nm. The values were plotted using Microsoft Excel software and RFU to nM conversion were calculated from the equation of the line.

### Introduction of point mutations into CHIKV and SFV *trans*-replicases

Point mutations, resulting in the following changes of encoded nsP2 protein, were made using T7-P1234 plasmid^39^ and PCR-based site directed mutagenesis; designations of the obtained constructs are provided in parentheses: Cys478 to Ala (T7-P12^CA^34), Trp479 to Ala (T7-P12^WA^34), Ser482 to Ala (T7-P12^SA^34) and Cys478 and Ser482 to alanine residues (T7-P12^CA+SA^34). To introduce similar mutations into SFV *trans-*replicase plasmid P123Z4^42^, hereafter designated as SFV-T7-P123^Z^4, PCR-based site directed mutagenesis was used. The obtained plasmids were designated as SFV-T7-P12^CA^3^Z^4, SFV-T7-P12^WA^3^Z^4, SFV-T7-P12^SA^3^Z^4 and SFV-T7-P12^CA+SA^3^Z^4. The sequences of the constructs were verified through DNA sequencing.

### *In vitro* transcription/translation and immunoprecipitation

*In vitro* transcription and translation were carried out using the TNT-coupled T7 rabbit reticulocyte lysate system (Promega) according to the manufacturer’s protocol. Reaction mixtures (50 μL) containing 20 μCi of [^35^S]methionine-cysteine mixture (Perkin-Elmer) and 2 μg of a T7-P1234, T7-P12^CA^34, T7-P12^WA^34, T7-P12^SA^34, T7-P12^CA+SA^34 or T7-P1234^GAA ^39 were incubated for 90 min at 30 °C; translation was stopped by adding cycloheximide to a final concentration of 1 mM. RNase A was added at a final concentration of 10 ng/μL, and the mixtures were incubated for another 5 min. Immunoprecipitation of CHIKV ns proteins and polyproteins was performed as previously described^16^ using rabbit polyclonal antisera against nsP1, nsP2, nsP3 and nsP4 (all in-house). The precipitated proteins were denatured by heating in Laemmli buffer, separated by SDS-PAGE, and visualized using a Typhoon imager (GE Healthcare).

### Analysis of CHIKV *trans-*replicase activity

Analysis of CHIKV *trans*-replicase activity was performed as previously described^39^. Briefly, BSR cells, grown on 35 mm plates to 90% confluence, were co-transfected with mixtures consisting of 1 μg of plasmid encoding wt or mutant CHIKV replicase and 1 μg of T7-Fluc-Gluc, a plasmid for expression of an RNA template^39^, using Lipofectamine LTX reagent according to the manufacturer’s (Invitrogen) protocols. At 18 h p.t. cells were lysed with *Renilla* luciferase (Rluc) assay lysis buffer (Promega); activities of Fluc and Gluc were analysed using the Dual-Luciferase Reporter Assay kit and Glomax SIS luminometer (Promega).

The levels of replicase-generated positive- and negative-strand RNAs present in transfected cells were analyzed by Northern blotting as previously described in ref. 46, with some modifications. Briefly, BSR cells co-transfected with 1 μg of plasmid encoding wt or mutant CHIKV replicase (T7-P1234, T7-P12^CA^34, T7-P12^WA^34, T7-P12^SA^34, T7-P12^CA+SA^34 or T7-P1234^GAA^) and 1.25 μg of T7-Rluc-Tom^39^ were lysed at 18 h p.t. with TRIsure reagent (Bioline), followed by RNA isolation according to the manufacturer’s instructions. Eight micrograms total RNA was fractionated on a denaturing 1% agarose gel and transferred to Hybond-N+ nylon filter (GE Healthcare). RNA was cross-linked to the membrane with Stratalinker (Stratagene). ^33^P-labeled RNA probes were made by *in vitro* transcription with T7 RNA polymerase from PCR-amplified DNA fragments containing sequences of the Rluc gene and the T7 promoter. The probe for positive-strand RNA detection corresponded to nucleotides 676 to 32, and the probe for negative-strand RNA detection corresponded to nucleotides 38 to 623 of the Rluc gene as present on template constructs. Hybridization was performed at 60 °C overnight, followed by several washes and 4 days exposure. Detection was performed using Typhoon Trio (GE Healthcare) instrument. Detection of positive- and negative-strand RNAs generated by SFV replicase was performed similarly except that Tmed^46^ was used as source of template RNA and plasmids SFV-T7-P123^Z^4, SFV-T7-P123^Z^4^GAA^, SFV-T7-P12^CA^3^Z^4^5^, SFV-T7-P12^WA^3^Z^4, SFV-T7-P12^SA^3^Z^4 and SFV-T7-P12^CA+SA^3^Z^4 were used to express different forms of SFV replicase.

### Construction, rescue and analysis of recombinant viruses

Mutations resulting in changes of Cys478 to Ala, Trp479 to Ala, Ser482 to Ala or Cys478 and Ser482 to alanine residues of nsP2 were introduced into pSP6-CHIKV (an infectious cDNA clone of CHIKV ECSA genotype, LR-2006-OPY1 isolate^47^); obtained clones were designated pSP6-CHIKV^C478A^, pSP6-CHIKV^W479A^, pSP6-CHIKV^S482A^ and pSP6-CHIKV^C478A+S482A^. All these manipulations were done by using PCR-based site-directed mutagenesis and subcloning procedures; sequences were verified.

Plasmids containing CHIKV cDNA were linearized and transcribed *in vitro* using the mMESSAGE mMACHINE SP6 Transcription kit (Ambion), to obtain capped RNAs. These procedures as well as ICA were performed as previously described^16^. Recombinant viruses (wt CHIKV and CHIKV^S482A^) were harvested at 18 h p.t.; for cells transfected with transcripts of pSP6-CHIKV^C478A^, pSP6-CHIKV^W479A^ or pSP6-CHIKV^C478A+S482A^ the cell culture supernatants were collected at 144 h p.t. The transfected cells were collected at the same time and lysed by boiling in Laemmli buffer.

### Western blot analysis

Cells transfected with 1 μg of T7-P1234, T7-P12^CA^34, T7-P12^WA^34, T7-P12^SA^34, T7-P12^CA+SA^34 or T7-P1234^GAA^ were lysed at 18 h p.t. with SDS loading buffer; lysates from cells transfected with RNA transcripts were obtained as described above. Proteins were separated by SDS-PAGE, transferred to nitrocellulose membranes, and detected using primary antibodies against CHIKV ns proteins (*trans*-replicase transfected cells) or against CHIKV capsid protein (RNA transcript transfected cells). Antibody against β-actin (sc-47778; Santa Cruz Biotechnology) was used as loading control. The membranes were then incubated with appropriate secondary antibodies conjugated to horseradish peroxidase (LabAs Ltd, Estonia) and proteins were visualized using ECL Immunoblot Detection kit (GE Healthcare).

### Metabolic labelling

Pulse-chase labelling of infected BHK-21 cells was carried out as described previously^15^. Briefly, BHK-21 cells were infected with wt CHIKV or CHIKV^S482A^ at a multiplicity of infection (MOI) of 20 PFU/cell. At 3 h post infection the cells were starved in methionine-cysteine-free DMEM (Life Technologies) for 30 min and then labelled with 50 μCi of [^35^S] methionine-cysteine mixture (Perkin-Elmer) for 15 min (pulse). In the chase samples, the pulse was followed by a chase for 15 or 60 min in medium containing an excess of unlabelled methionine and cysteine. The cells were then lysed by boiling in 1% SDS, proteins in the cell lysates were immunoprecipitated using combinations of antibodies (nsP1 + nsP3 and nsP2 + nsP4), separated by SDS-PAGE, and visualized using a Typhoon imager (GE Healthcare).

### Electron microscopy

BHK-21 cells were seeded onto 35 mm glass-bottom dishes (P35G–1.5-14-C; MatTek). The next day, cells were infected with wt CHIKV or CHIKV^S482A^ at MOI 10 PFU/cell. 8 h post infection, the supernatant was discarded and cells were fixed with 2% glutaraldehyde in 0.1 M sodium cacodylate buffer for 30 min at room temperature and washed in the same buffer. Sample preparation for transmission electron microscopy was done as described earlier^48^. Sections were imaged in JEOL JEM-1400 electron microscope operated at 80 kV and images were acquired by a bottom-mounted camera Gatan Orius SC 1000B (Gatan Inc.).

## Additional Information

**How to cite this article**: Rausalu, K. *et al.* Chikungunya virus infectivity, RNA replication and non-structural polyprotein processing depend on the nsP2 protease’s active site cysteine residue. *Sci. Rep.* **6**, 37124; doi: 10.1038/srep37124 (2016).

**Publisher’s note:** Springer Nature remains neutral with regard to jurisdictional claims in published maps and institutional affiliations.

## Supplementary Material

Supplementary Information
