# Supplementary material for: Chikungunya virus infectivity, RNA replication and non-structural polyprotein processing depend on the nsP2 protease’s active site cysteine residue
Source: Sci Rep. 2016 Nov 15;6:37124. doi: 10.1038/srep37124 (PMC5109220; doi:10.1038/srep37124)

Supplementary Information

Chikungunya virus infectivity, RNA replication and non-structural polyprotein processing depend on the nsP2 protease's active site cysteine residue.

Kai Rausalu, Age Utt, Tania Quirin, Finny S. Varghese, Eva Žusinaite, Pratyush Kumar Das,

Tero Ahola, Andres Merits.

In this file full-length images, used to generate Figures 4**b**, 5**b**, 6**b**, 6**c** and 7**b**, are provided. Parts of images, used on final Figures, are boxed.

Figure 4b. C478A substitution in nsP2 region blocks the processing of CHIKV P1234 in cell-free system (full images).

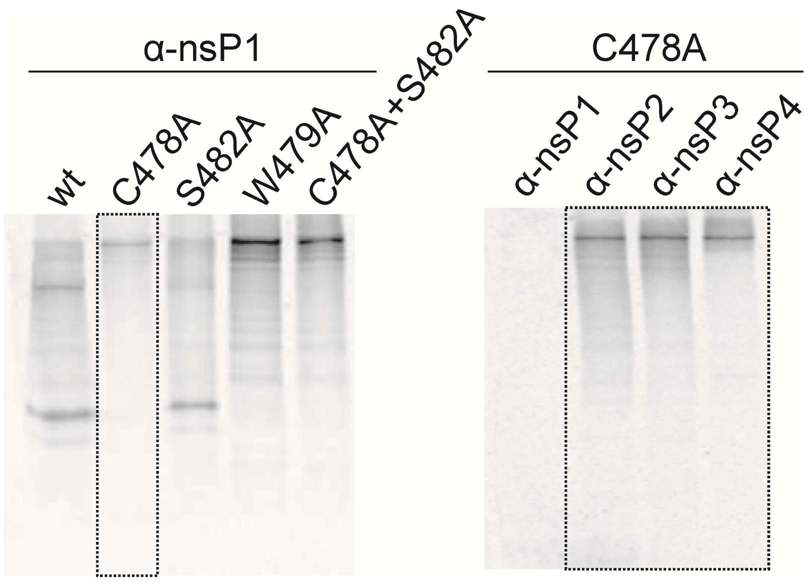

Figure 5b. C478A and W479A substitutions in nsP2 region abolish processing of P1234 in transfected cells

Western blot obtained using antibody against nsP1 (same gel, two different exposures)

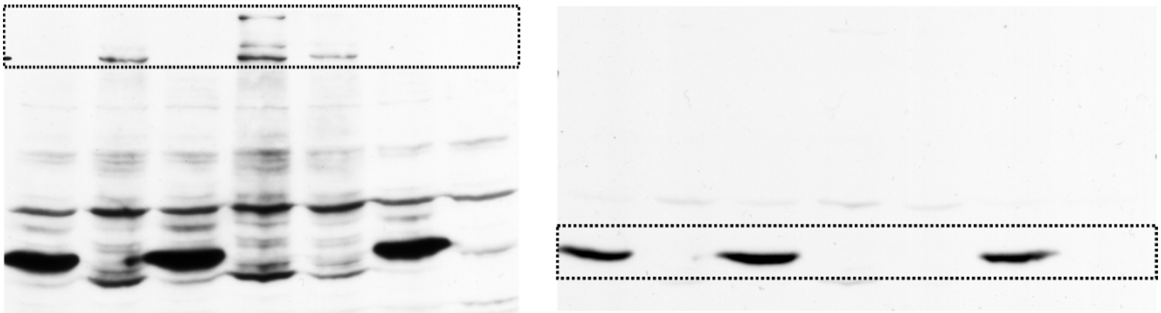

Western blot obtained using antibody against nsP2 (same gel, two different exposures)

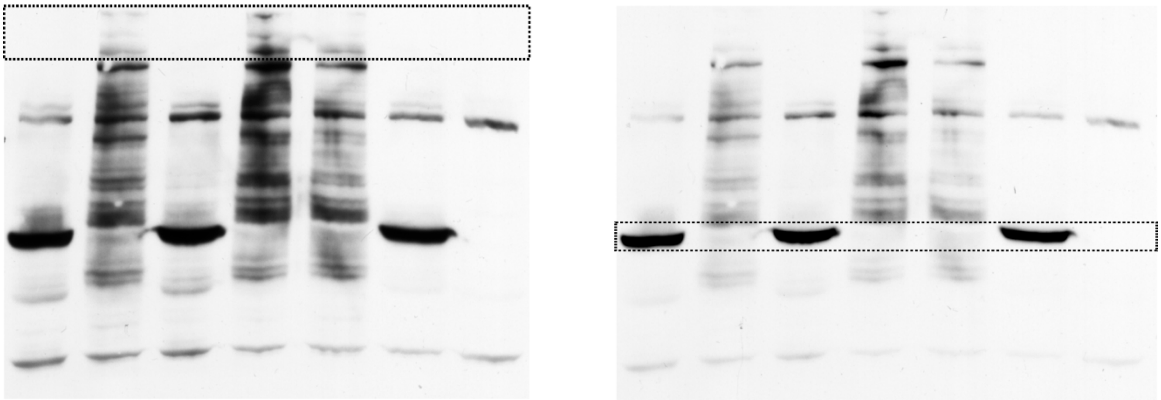

Western blot obtained using antibody against nsP3 (same gel, two different exposures)

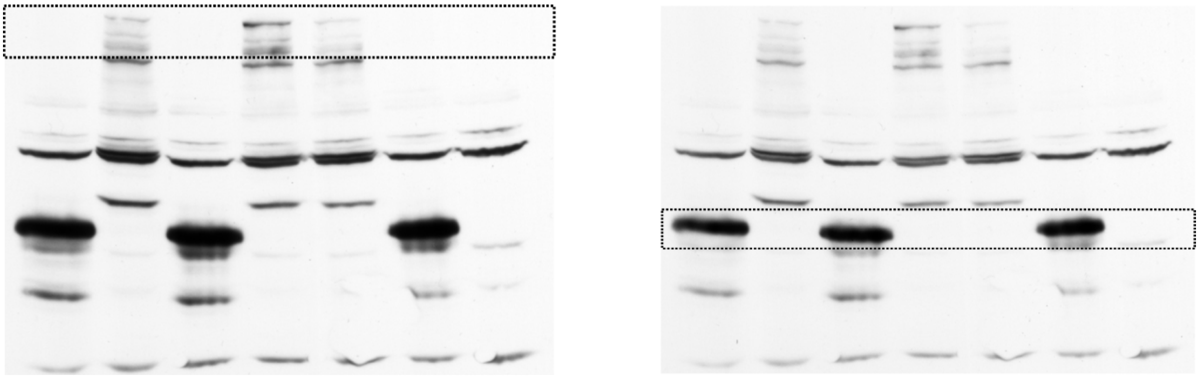

Western blot obtained using antibody against nsP4 (same gel, two different exposures)

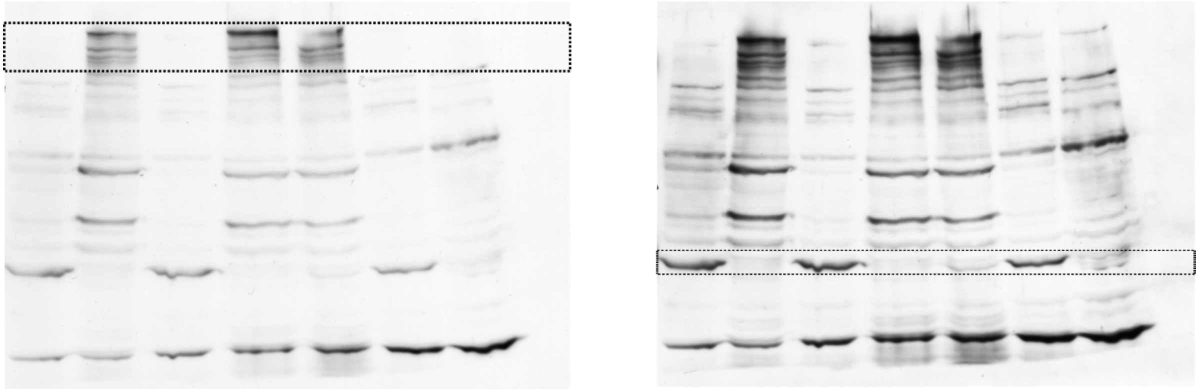

Figure 6b. Synthesis of negative-strand RNAs by SFV and CHIKV *trans*-replicases (full-length blot)

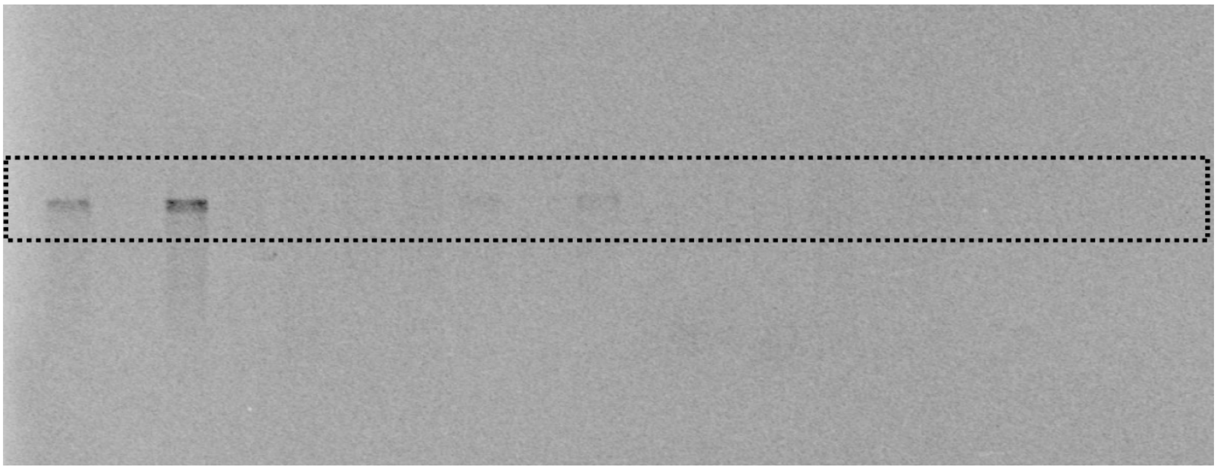

Figure 6c. Synthesis of positive-strand RNAs by SFV and CHIKV *trans*-replicases (full-length blot)

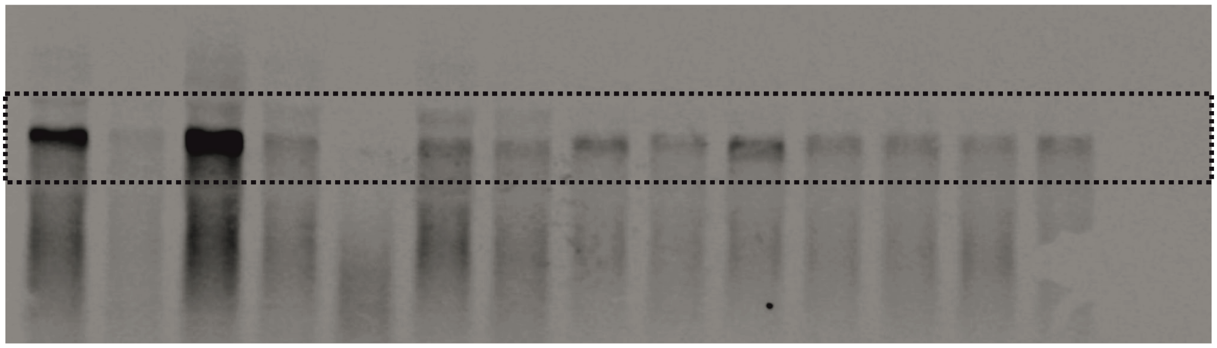

Figure 7b. Western blot obtained using antibody against capsid protein. Two different exposures of the same gel are shown

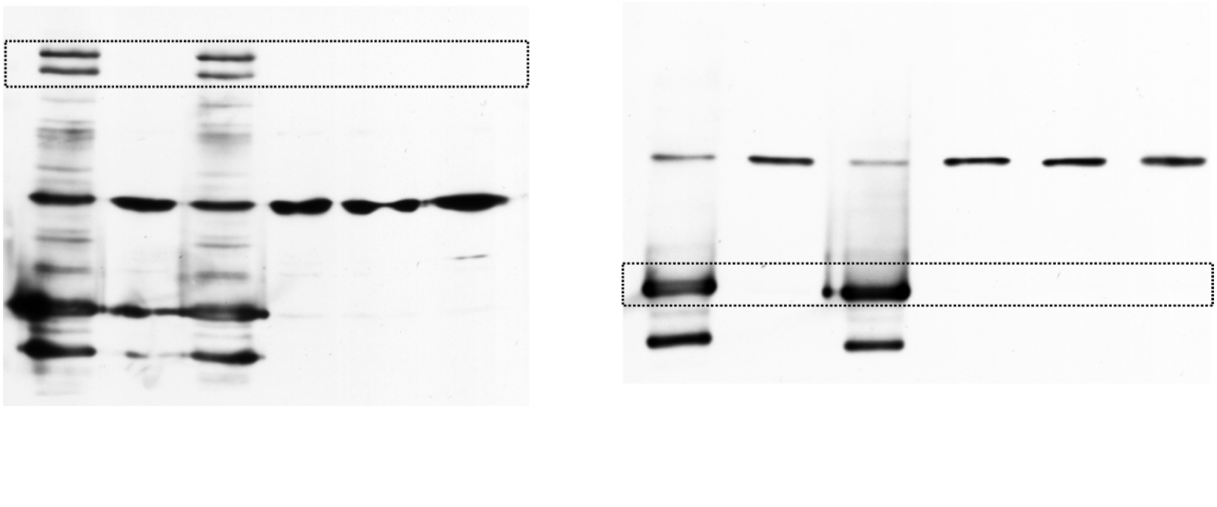

Supplement: Supplementary Information [file srep37124-s1.pdf]
